# A Cadaveric Study on the Variations in the Infraclavicular Part of Brachial Plexus in Axillary Region and Upper Arm

**DOI:** 10.1155/bmri/6650184

**Published:** 2025-10-31

**Authors:** E. Bozhikova, J. C. Davis, P. H. Moon, N. Uzunov

**Affiliations:** ^1^ Department of Biomedical Sciences, Mercer University School of Medicine, Columbus, Georgia, USA, mercer.edu; ^2^ Medical Student, Mercer University School of Medicine, Columbus, Georgia, USA, mercer.edu; ^3^ Department of Maxillofacial Surgery, University Hospital “Medika”, Ruse, Bulgaria

**Keywords:** anatomical variations, axilla, axillary nerve, brachial plexus, brachial plexus variations, median nerve, musculocutaneous nerve, radial nerve, surgical anatomy

## Abstract

**Background:**

The brachial plexus shows frequent anatomical variations that can complicate diagnosis, surgical planning, and anesthetic procedures. Detailed knowledge of these variations is essential to prevent iatrogenic injury and improve clinical outcomes.

**Materials and Methods:**

Eighteen donors (36 upper limbs) were dissected at Mercer School of Medicine, Columbus, Georgia, United States. The sample included 10 males and 8 females, aged 36–89 years (mean age: 67.67 years). Dissections followed *Grant′s Dissector*, 17th edition, with all variations in the infraclavicular brachial plexus and its branches in the axillary region and upper arm documented, photographed, and measured.

**Results:**

Variations were found in 16 brachial plexuses (44.4%) from nine donors. In total, 32 variations were identified, involving the medial and posterior cords, median, musculocutaneous, ulnar, radial, axillary, lower subscapular, and thoracodorsal nerves. Bilateral variations were more common (77.8%) than unilateral ones (22.2%), often with two to three variants on one side. Several rare or previously undescribed findings were noted, including a median nerve with an accessory lateral root located between the main and aberrant axillary arteries, a rare communication between the medial cord and lateral root of the median nerve, a rare communication between the ulnar and median nerves in the upper arm, bifurcation of the musculocutaneous nerve, trifurcation of the radial nerve, trifurcation of the axillary nerve, posterior division of the axillary nerve passing through the triangular space, posterior division of the axillary nerve supplying both teres minor and teres major, bifurcated axillary nerve accompanied by an accessory subscapularis muscle, accessory thoracodorsal nerve arising from the axillary nerve, and quadfurcation of the posterior cord.

**Conclusion:**

Contrary to previous literature, axillary nerve variations were the most common. Bilateral deviations were frequent, often associated with arterial variations. Multiple rare and undescribed patterns identified in this study expand current knowledge of infraclavicular brachial plexus anatomy.

**Summary**

• This study documents several infraclavicular brachial plexus patterns not previously described in the literature, offering new insights with direct clinical relevance for surgical and anesthetic procedures.

## 1. Introduction

**Figure 1:**
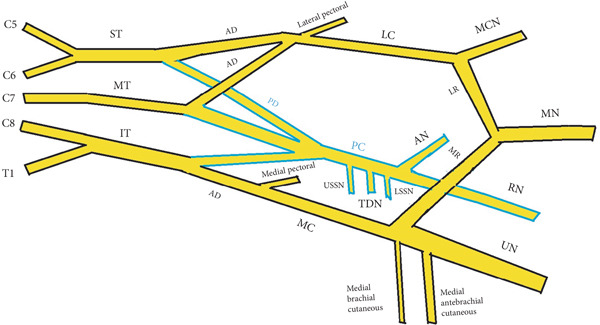
Schematic illustration of the normal infraclavicular brachial plexus anatomy. Abbreviations: AD, anterior division; AN, axillary nerve; IT, inferior trunk; LC, lateral cord; LR, lateral root of the median nerve; LSSN, lower subscapular nerve; MC, medial cord; MCN, musculocutaneous nerve; MN, median nerve; MR, medial root of the median nerve; MT, middle trunk; PC, posterior cord; PD, posterior division; RN, radial nerve; ST, superior trunk; TDN, thoracodorsal nerve; UN, ulnar nerve; USSN, upper subscapular nerve.

The brachial plexus (BP) is the main nerve supply to the upper limb. According to the classical description, the plexus is typically formed by the anterior rami of C5–T1, which join in the posterior neck to form the superior (C5–C6), middle (C7), and inferior (C8–T1) trunks. Each trunk divides into anterior division (AD) and posterior division (PD). The ADs of the superior trunk (ST) and middle trunk (MT) unite to form the lateral cord (LC), the AD of the inferior trunk (IT) continues as the medial cord (MC), and the PDs of all three trunks form the posterior cord (PC). The cords surround the axillary artery (AA) in the axilla and give rise to major peripheral nerves. The LC gives off the lateral pectoral nerve, the musculocutaneous nerve (MCN), and the lateral root (LR) of the median nerve (MN). The MC gives rise to the medial pectoral nerve, the medial cutaneous nerves of the arm and forearm, the ulnar nerve (UN), and the medial root (MR) of the MN. The branches of the PC are the upper subscapular nerve (USSN) and lower subscapular nerve (LSSN), thoracodorsal nerve (TDN), axillary nerve (AN), and radial nerve (RN) (Figure 1) [1].

The BP is notable for its frequent anatomical variations and vulnerability to injuries. Over half of all peripheral nervous system variations occur within the BP, impacting diagnostic accuracy, treatment planning, and surgical outcomes [2–6]. A detailed knowledge of these variations is essential for reconstructive surgery, regional anesthesia, and radiological evaluation of peripheral nerves [6–8]. They are also important for understanding nerve dysfunctions, conducting neurophysiological studies, interpreting motor and sensory deficits, achieving correct diagnosis, planning effective treatments, and reducing the risk of iatrogenic injuries [9–15].

BP injuries can result from trauma, obstetric traction, or surgical procedures, leading to severe functional loss and reduced quality of life [6, 16]. Procedures in the axilla and upper arm—including nerve blocks, tumor resections, and lymphadenectomies—carry higher risks when variations are present [2, 6, 17, 18]. Unrecognized variations may lead to failed regional anesthesia, misdiagnoses, or postoperative nerve or vascular injuries [6, 19–21].

Variations in nerve branching of BP are clinically relevant in treating humeral fractures, diagnosing entrapment syndromes, and planning surgical approaches [10, 15]. They are especially important in microsurgery, nerve transfers, and neurotization procedures, where precise anatomical knowledge improves patient outcomes [6, 12].

Despite many publications, most BP variation studies are limited to isolated case reports [5]. The present study investigates variations in the infraclavicular BP in the axilla and upper arm in the US population. The US population represents a diverse mix of ethnic backgrounds, potentially encompassing a broader spectrum of anatomical variations than more homogenous populations. This diversity increases the relevance of our findings for varied clinical and surgical contexts.

## 2. Materials and Methods

This study examined 18 donors (36 upper limbs) at Mercer School of Medicine, Columbus, Georgia, United States. The sample included 10 males and 8 females, aged 36–89 years (mean age: 67.67 years). Donors were preserved in a solution containing ethyl alcohol (4.5%), glycerol (0.5%), phenol (1%), phenoxyethanol (0.5%), and water (93.5%).

The sample size was comparable to or exceeded that of previous cadaveric studies on BP variations, which typically range from 12 to 30 donors [2, 6, 8, 22–24]. No universally accepted minimum exists for such anatomical studies; however, prior works with similar or smaller samples have reported meaningful variation patterns. Our sample was therefore considered adequate to detect and document both common and rare variants.

Inclusion criteria required intact BP and branches without pathological changes. Dissections were performed following *Grant′s Dissector*, 17th edition [25], focusing on the BP and its branches in the axillary region and upper arm. All variations were documented and photographed.

Measurements were taken with a RoHS electronic caliper (KWB) with an accuracy of 0.01 mm and a measurement speed of 1.5 m/s, powered by a 1.45 V battery. Linear dimensions were recorded in millimeters.

Statistical analysis was performed using IBM SPSS Statistics for Windows, Version 27 (IBM Corp., Armonk, New York). Descriptive statistics were applied to summarize frequencies and age distribution. The chi‐square test (*χ* ^2^) was used to assess associations between the presence of variations and donor sex or side. A *p* value < 0.05 was considered statistically significant.

## 3. Results

Variations were found in nine donors (six males and three females). Sixteen BPs (44.4%) showed at least one variation. Across these, 32 variable nerve structures (88.9%) were identified. Some nerves had more than one variation, resulting in 36 documented variations overall. Bilateral variations occurred in seven donors (77.8%) and were significantly more common than unilateral variations (22.2%). In most arms, two or three variations were present on a single side. No statistically significant association was found between the presence of variations and donor sex or side.

The affected structures were distributed as follows: MC, one case (3.1%); PC, three cases (9.4%); MN, four cases (12.5%); MCN, six cases (18.8%); UN, one case (3.1%); RN, five cases (15.6%); AN, nine cases (28.1%); LSSN, one case (3.1%); and TDN, two cases (6.2%).

The following novel or rare variations were found:

• MN with an accessory LR located between the main and aberrant AA—to our knowledge, this specific configuration has not been previously described.
• An uncommon communication between the MC and the LR of the MN crossing anterior to the AA—to our knowledge, only one similar case has been reported.
• MCN bifurcation into distinct lateral division (LD) and medial division (MD) after giving a communicating branch (CB) to the MN, with both divisions supplying arm flexors—no identical case found in the literature.
• Very distal CB between MCN and MN, measuring over 100 mm in length and joining near the cubital fossa—unusually long trajectory compared to most reported cases.
• CB between the UN and MN in the upper arm—while well‐known distally, this proximal occurrence is distinctly uncommon.
• Trifurcation of the RN above the triangular interval (TrI) and radial groove—previously only reported within the radial groove; our cases differ in occurring proximal to both.
• PD of AN passing through the triangular space (TrSp) together with the circumflex scapular artery (CSA)—to our knowledge, this has not been previously reported.
• PD of AN supplying both teres minor (TMi) and teres major (TMj)—to our knowledge, this is the first description of this innervation pattern.
• Trifurcation of the AN, with the AD and a thin middle branch entering the quadrangular space (QdrSp), and the PD passing through the TrSp—to our knowledge, this specific branching pattern has not been previously reported.
• Combination of AN variation with accessory subscapularis muscle (SSM) and associated arterial variation—to our knowledge, not previously described.
• Accessory TDN originating from the AN—previously reported only from the PC.
• Quadfurcation of the PC into AN, RN, TDN, and LSSN—to our knowledge, this has not been previously described.

### 3.1. MN Formation Patterns

**Figure 2:**
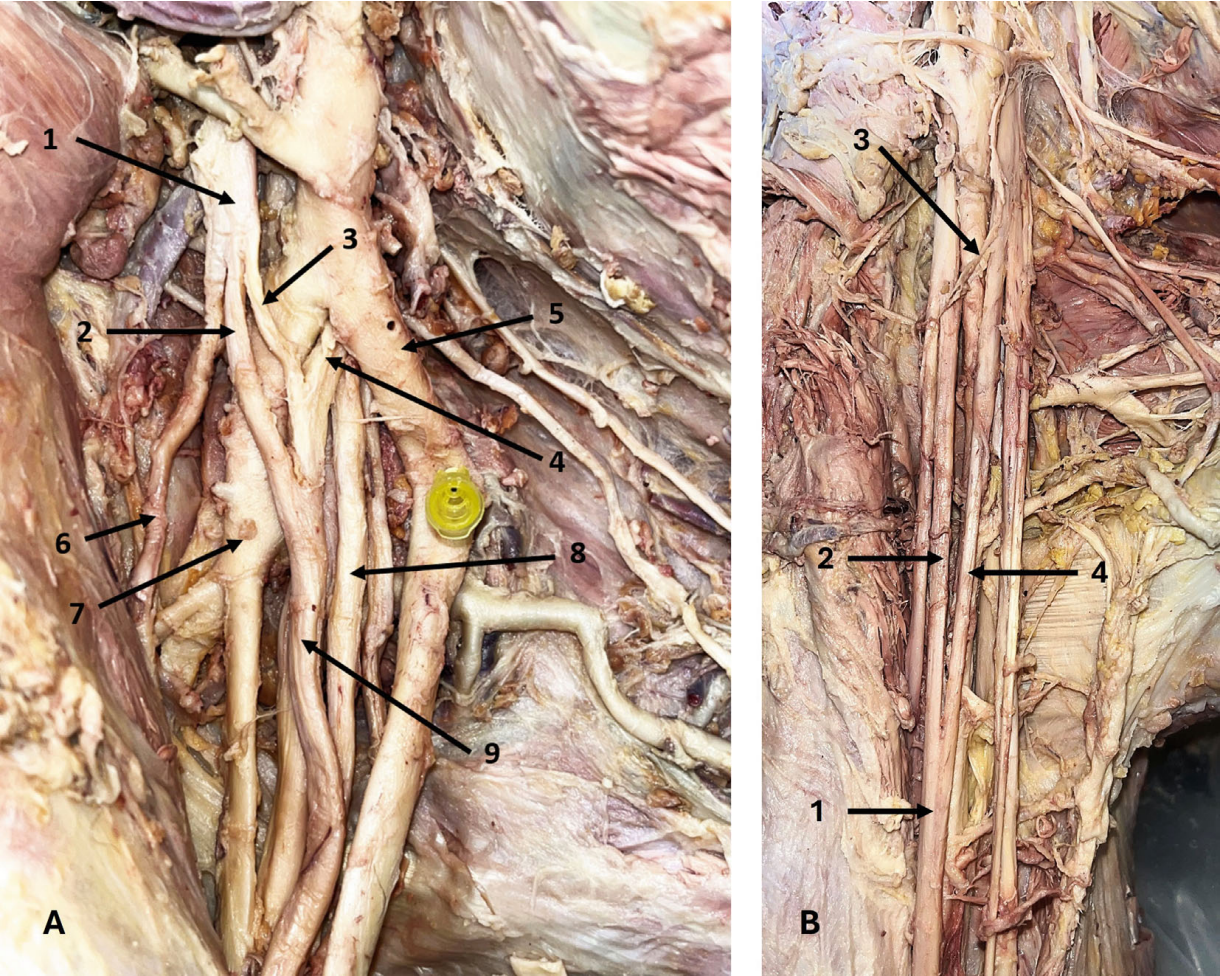
Variations of the MN. (A) An accessory LR of the MN (right axilla): 1, LC; 2, LR of MN; 3, accessory LR of MN; 4, MR of MN; 5, main AA; 6, MCN; 7, aberrant AA; 8, UN; 9, MN. (B) Low fusion of the roots of the MN and CB between the MC and LR of MN (right axilla): 1, MN; 2, MR of the MN; 3, CB between the MC and LR of the MN; 4, LR of MN. Abbreviations: AA, axillary artery; CB, communicating branch; LC, lateral cord; LR, lateral root; MC, medial cord; MCN, musculocutaneous nerve; MN, median nerve; MR, medial root; UN, ulnar nerve.

Three MNs had an accessory root originating from the LC of the BP. One represented a rare configuration in the right axilla of a 58‐year‐old male donor, with the MN positioned anterior to an aberrant AA and posterior to the main AA (Figure 2A)—to our knowledge, this configuration has not been previously reported. The other two accessory roots were observed in the right axilla of a 65‐year‐old male donor and in the left axilla of a 78‐year‐old male donor.

Another MN variation was found in the right arm of a 36‐year‐old male donor and was associated with a MC variation. In this case, the MN roots merged at a lower level—within the proximal one‐third of the upper arm, 75.64 mm distal to the origin of the MCN. Additionally, the MC gave a CB to the LR of the MN, located anterior to the AA (Figure 2B)—an uncommon communication previously reported only once in the literature.

### 3.2. MCN Variations

Six MCNs showed anatomical variations, with 10 variations in total. Three did not pierce the coracobrachialis muscle (CBM). In these cases, the CBM was supplied by a thin branch from the LC.

**Figure 3:**
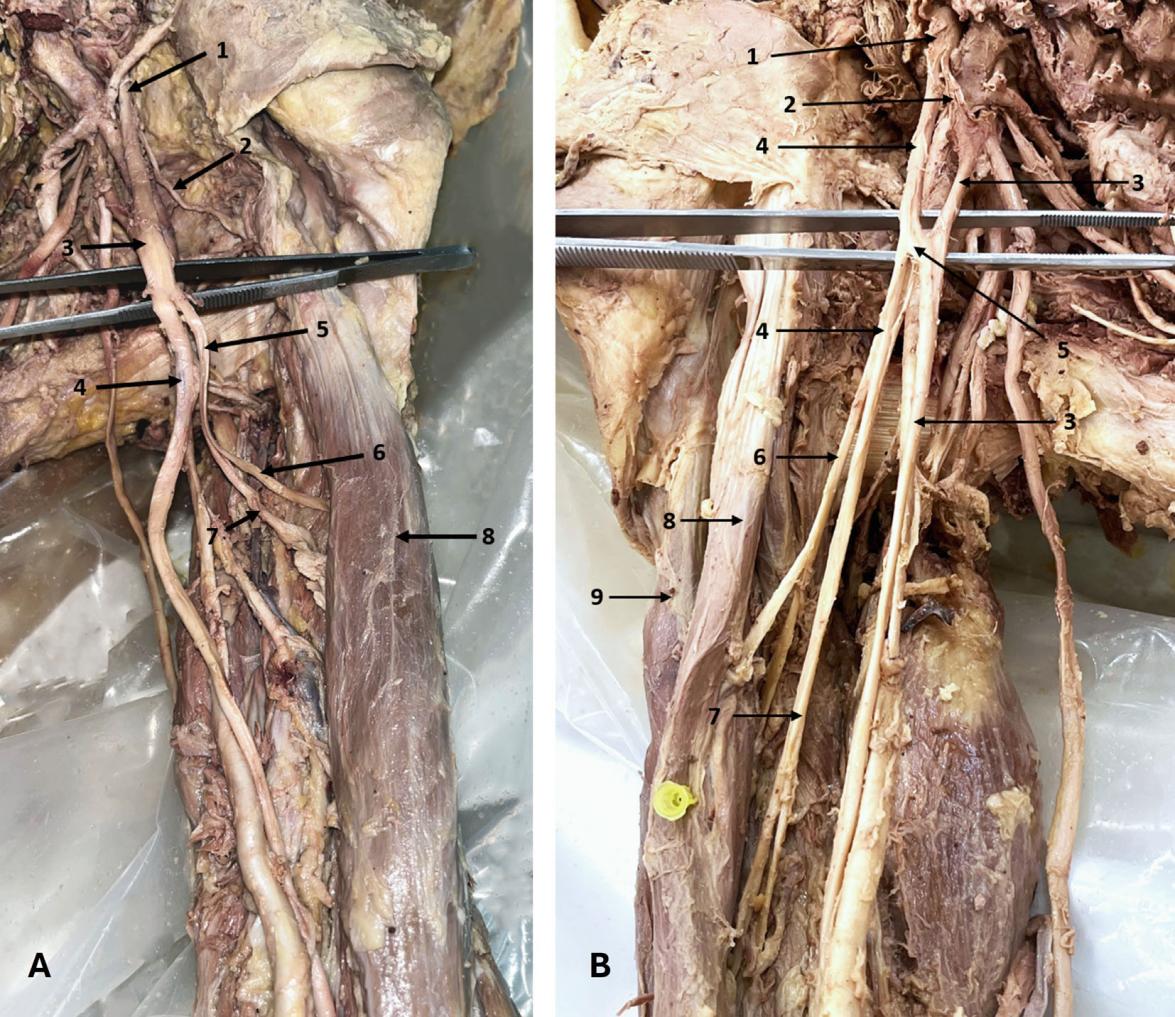
Variations of the MCN. (A) Fusion of the MCN with the MN (left arm): 1, LC; 2, branch to CBM; 3, fused MCN and MN; 4, MN; 5, MCN; 6, branch to BBM; 7, MCN; 8, BBM. (B) Bifurcation of the MCN (right arm): 1, LC; 2, LR of MN; 3, MN; 4, MCN; 5, CB from MCN to MN; 6, lateral division of MCN; 7, medial division of MCN; 8, long head of BBM; 9, short head of BBM. Abbreviations: BBM, biceps brachii muscle; CB, communicating branch; CBM, coracobrachialis muscle; LC, lateral cord; LR, lateral root; MCN, musculocutaneous nerve; MN, median nerve.

In a 55‐year‐old male donor, the left MCN fused with the MN, branching from it 29.18 mm distal to MN formation. It then supplied the biceps brachii muscle (BBM), entered the space between the BBM and brachialis muscle (BM), gave a branch to the BM, and continued as the lateral cutaneous nerve of the forearm (LCNFA) (Figure 3A). On the right side of the same donor, the MCN did not pierce the CBM and gave a CB to the MN 70.61 mm distal to its origin. Following this, the MCN bifurcated into LD and MD—a branching pattern not previously described. The LD supplied the BBM and BM, while the MD entered the space between the BBM and BM, gave a branch to the BM, and continued as the LCNFA (Figure 3B). On both sides, the CBM was supplied by the LC.

Two other MCNs that did not pierce the CBM were found bilaterally in a 78‐year‐old donor. Both gave CBs to the MN.

### 3.3. CBs Between MCN and MN

**Figure 4:**
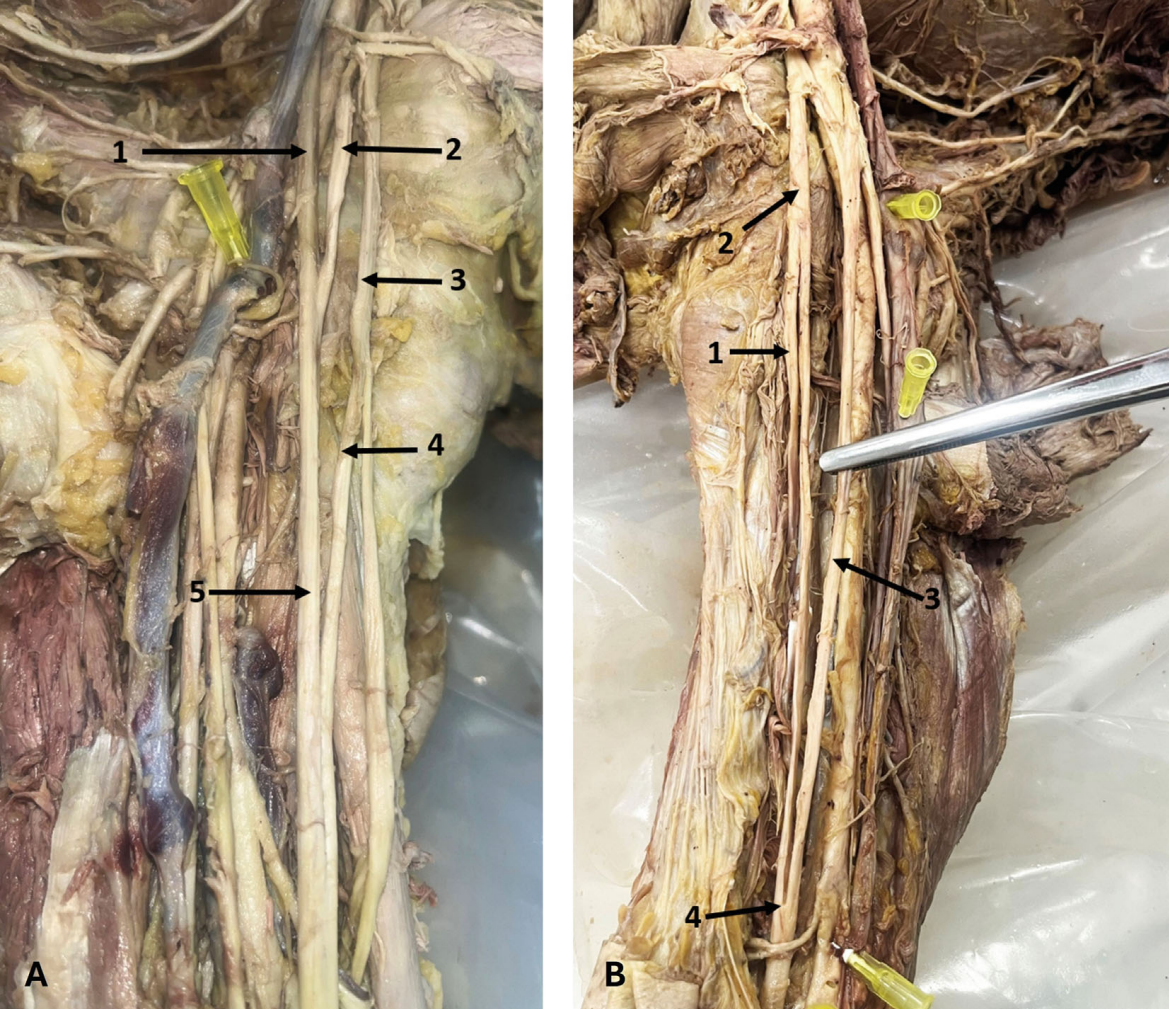
Variations of the MCN. (A) CB between the MCN and MN in the middle third of the arm (left arm): 1, MR of MN; 2, LR of MN; 3, MCN; 4, CB between the MCN and MN; 5, MN. (B) CB between the MCN and MN in the distal third of the arm (right arm): 1, MCN; 2, MN; 3, CB between the MCN and MN; 4, MCN. Abbreviations: CB, communicating branch; LR, lateral root; MCN, musculocutaneous nerve; MN, median nerve; MR, medial root.

Five CBs between the MCN and MN were identified. Three originated from MCNs that did not pierce the CBM, and two were located proximal to the MCN entry into the CBM. Two CBs were in the upper third of the arm, two in the middle third (Figure 4A), and one in the distal third (Figure 4B). The distal CB was in the right arm of a 78‐year‐old female donor. It arose 36.47 mm distal to the MCN origin, which did not pierce the CBM, and joined the MN near the cubital fossa. This branch measured 107.81 mm in length and represents a notably long CB trajectory compared to most reported cases.

### 3.4. CB Between UN and MN

**Figure 5:**
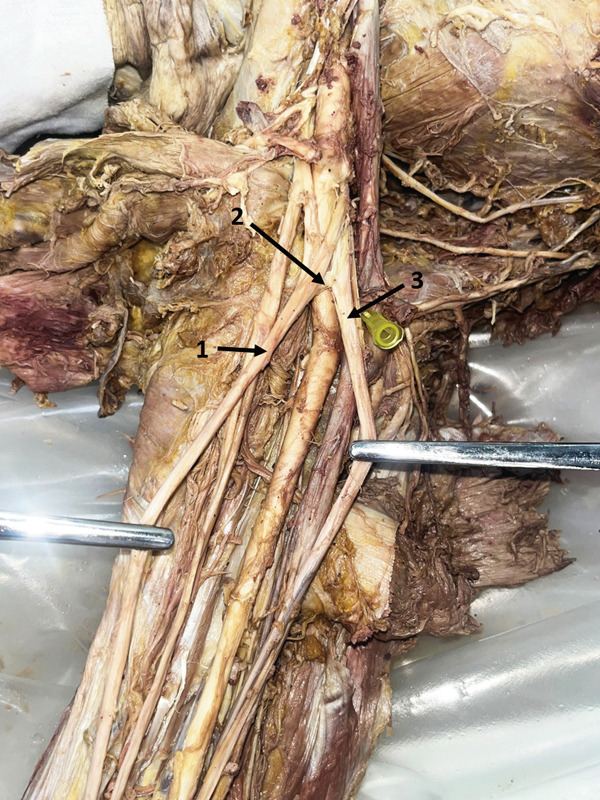
Communicating branch between the UN and MN (right arm): 1, MN; 2, CB; 3, UN. Abbreviations: CB, communicating branch; MN, median nerve; UN, ulnar nerve.

In the right arm of a 78‐year‐old female donor, the UN gave a CB to the MN. This branch originated 13.75 mm distal to the UN formation (Figure 5)—a rare upper arm connection between these nerves.

### 3.5. RN Variations: High Division of the RN

**Figure 6:**
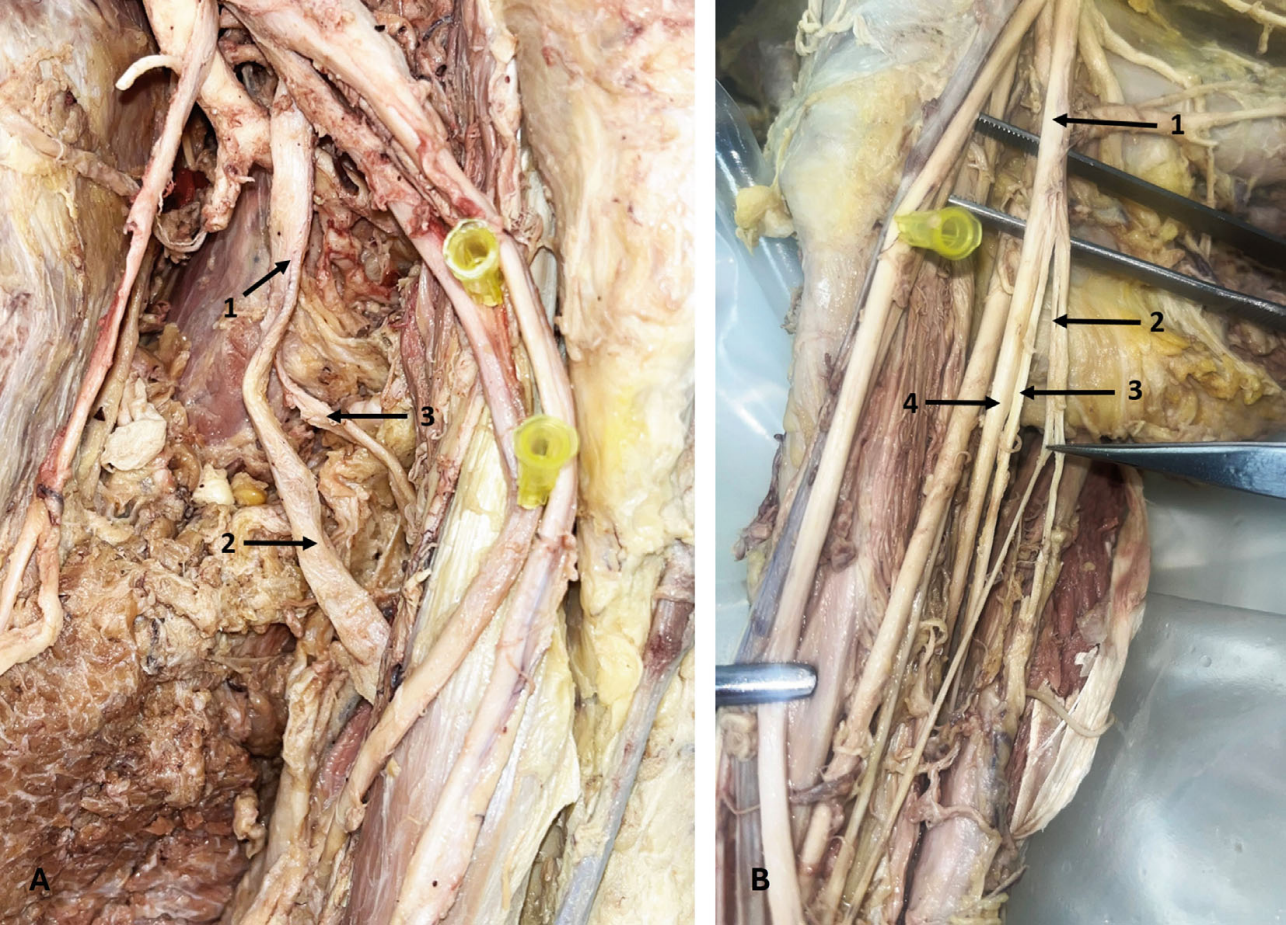
Variations of the RN. (A) Bifurcation of the RN (left arm): 1, RN; 2, anterior division; 3, posterior division to TrM. (B) Trifurcation of the RN (right arm): 1, RN; 2, medial division to TrM; 3, middle division; 4, lateral division. Abbreviations: RN, radial nerve; TrM, triceps muscle.

Five RN variations were observed, all involving a high division of the nerve. In three arms, the RN divided into two branches—either AD and PD or MD and LD. The PD or MD supplied the triceps muscles (TrM), while the other division continued as the main RN (Figure 6A). In two arms, the RN trifurcated into lateral, middle, and medial branches. The lateral and middle branches entered the TrI, while the MD supplied the TrM (Figure 6B)—a rare finding not described in the literature.

### 3.6. AN Variations: High Division of the AN

**Figure 7:**
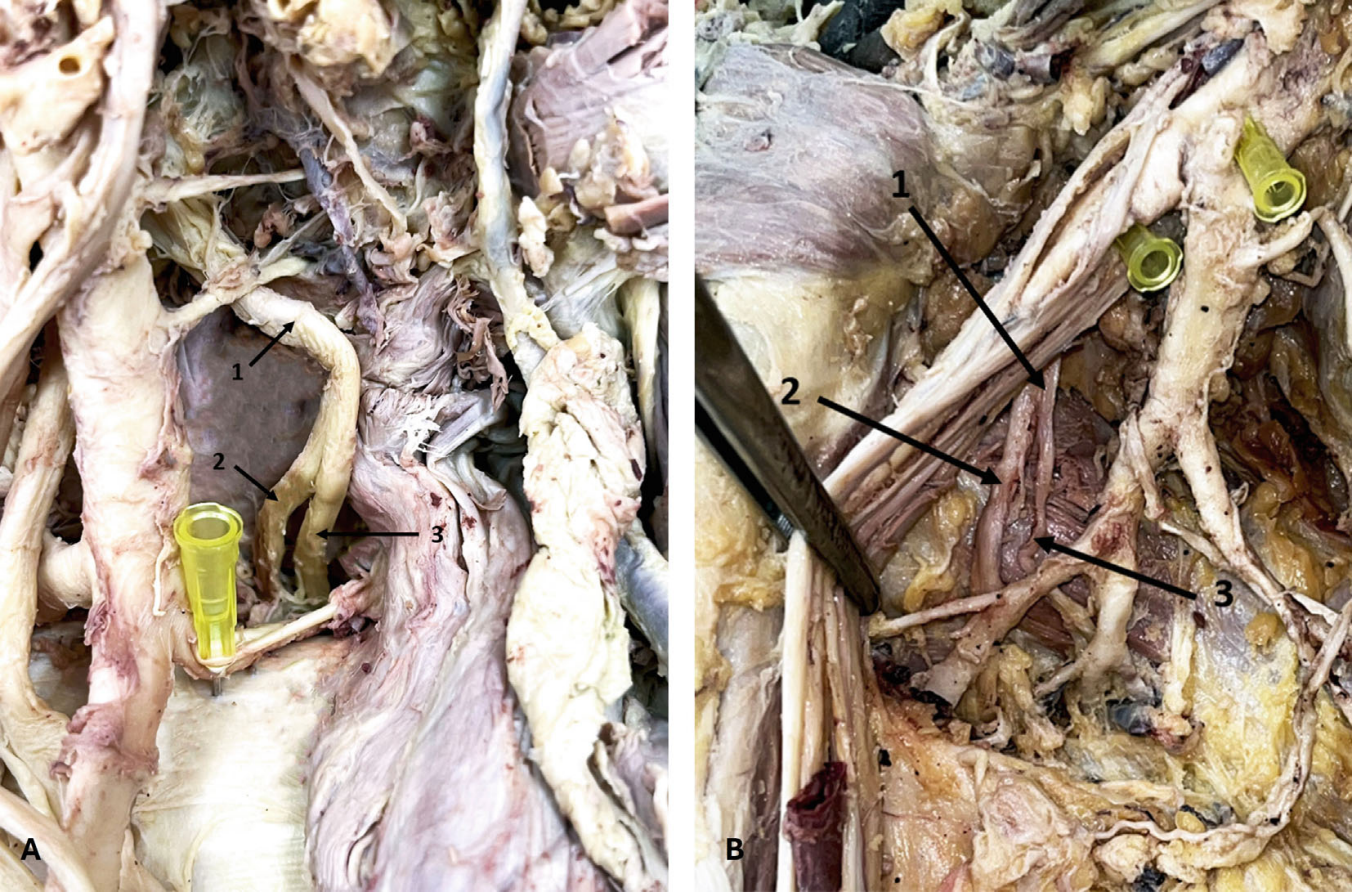
Variations in the AN. (A) Bifurcation of the AN (left axilla): 1, AN; 2, AD; 3, PD. (B) Bifurcation of the AN (right axilla): 1, AN; 2, AD; 3, PD and circumflex scapular artery passing through the triangular space. Abbreviations: AD, anterior division; AN, axillary nerve; PD, posterior division.

**Figure 8:**
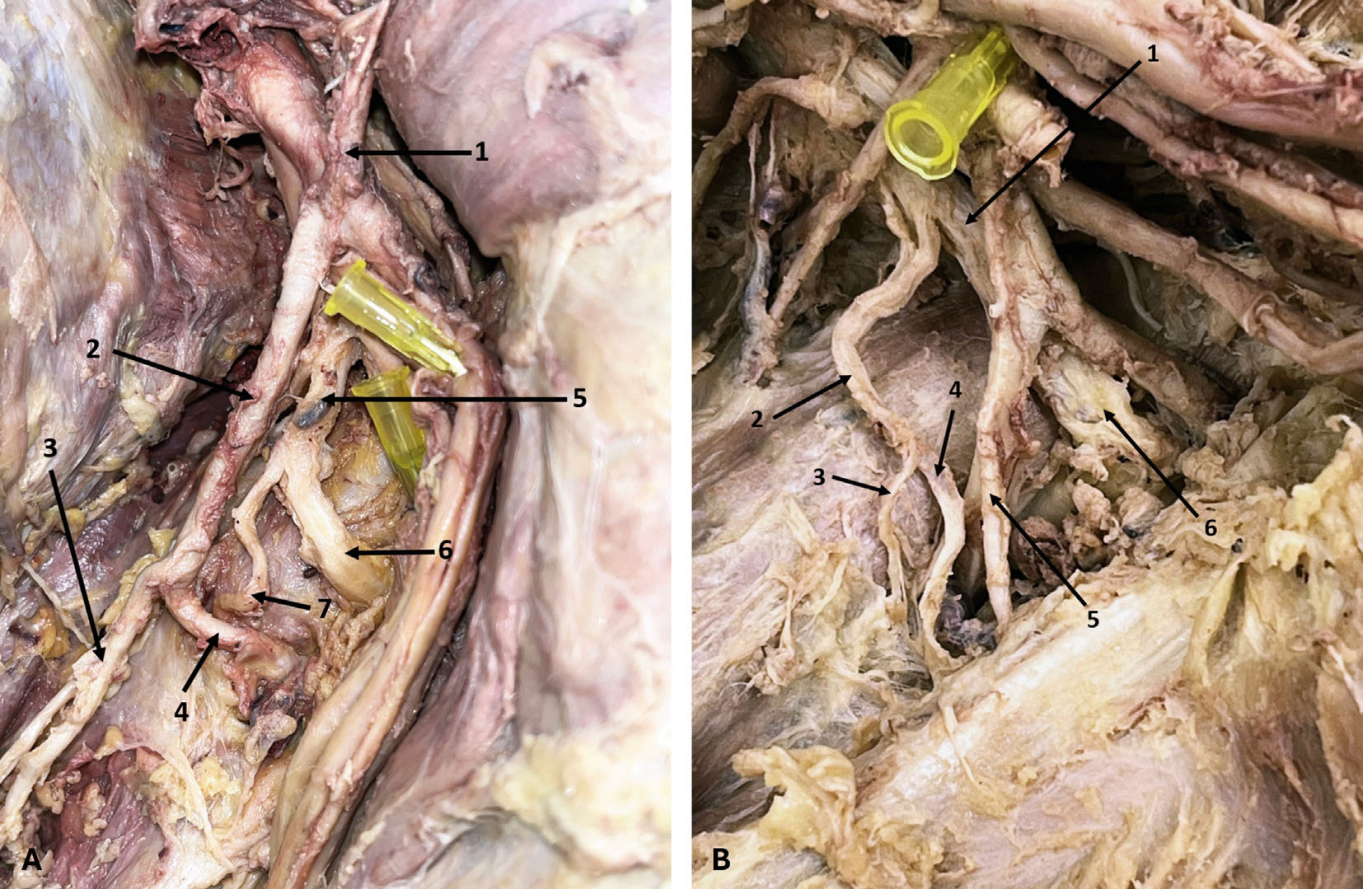
Variations in the AN. (A) PD of the AN supplying teres minor (left axilla): 1, thoracoacromial artery; 2, subscapular artery; 3, thoracodorsal artery; 4, circumflex scapular artery; 5, AN; 6, AD; 7, PD to teres minor. (B) PD of the AN supplying both teres minor and teres major (left axilla): 1, AN; 2, PD; 3, branch to teres minor; 4, branch to teres major; 5, branch from CSA to teres major; 6, AD. Abbreviations: AD, anterior division; AN, axillary nerve; CSA, circumflex scapular artery; PD, posterior division.

Nine ANs showed high bifurcation into AD and PD proximal to the QdrSp. In three arms, both divisions entered the QdrSp (Figure 7A). In two cases, the PD passed through the TrSp alongside the CSA (Figure 7B)—a course not previously documented. In two cases, the PD bifurcated, sending branches to TMi and TMj (Figure 8A)—to our knowledge, this is the first report of the PD innervating both muscles. In two arms, the PD supplied only TMi (Figure 8B).

**Figure 9:**
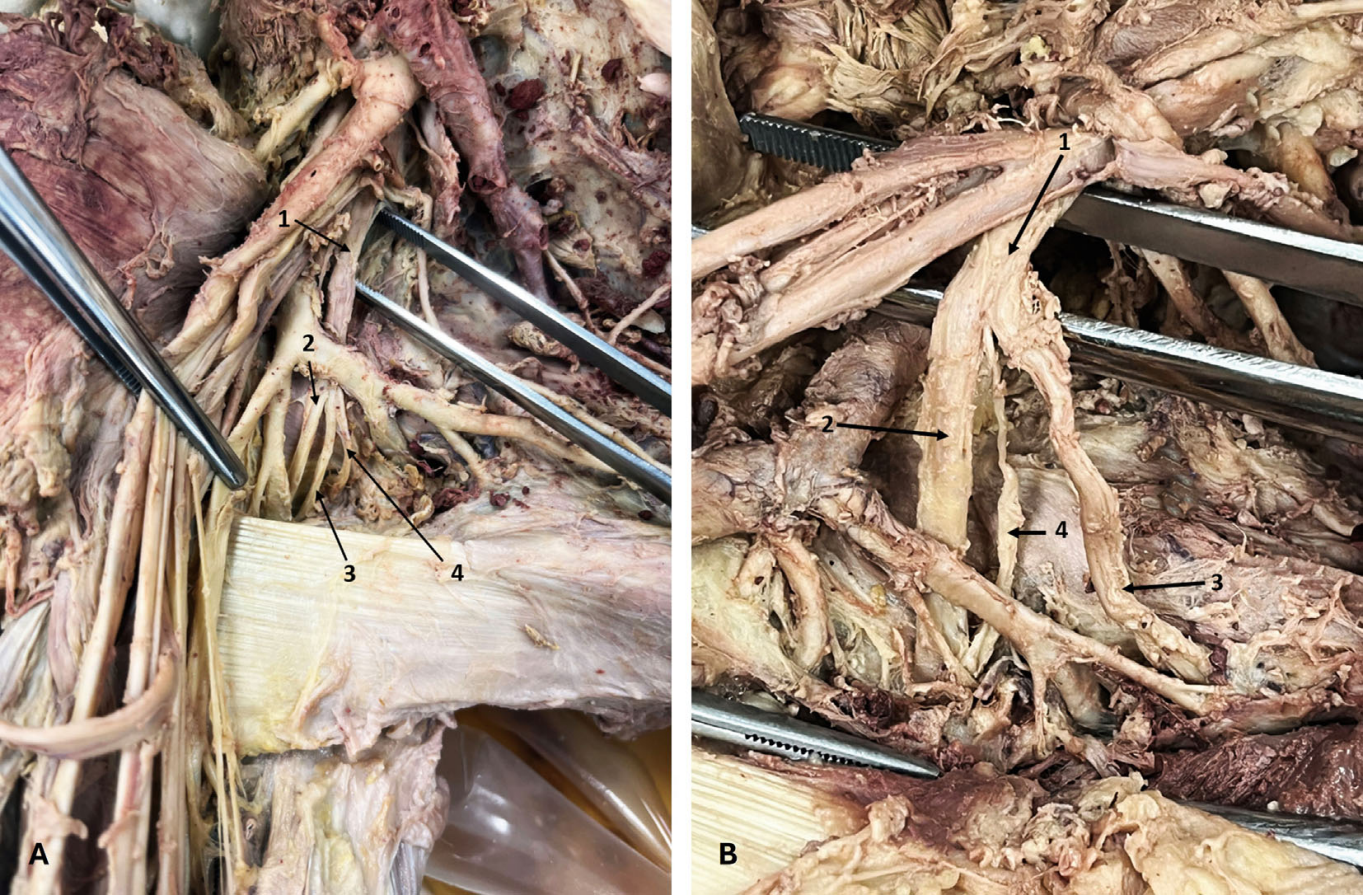
Variations in the AN. (A) Trifurcation of the AN (right axilla): 1, AN; 2, AD; 3, PD; 4, posterior branch of PD. (B) Trifurcation of the AN with the PD passing through the triangular space (right axilla): 1, AN; 2, AD; 3, PD; 4, middle branch. Abbreviations: AD, anterior division; AN, axillary nerve; PD, posterior division.

**Figure 10:**
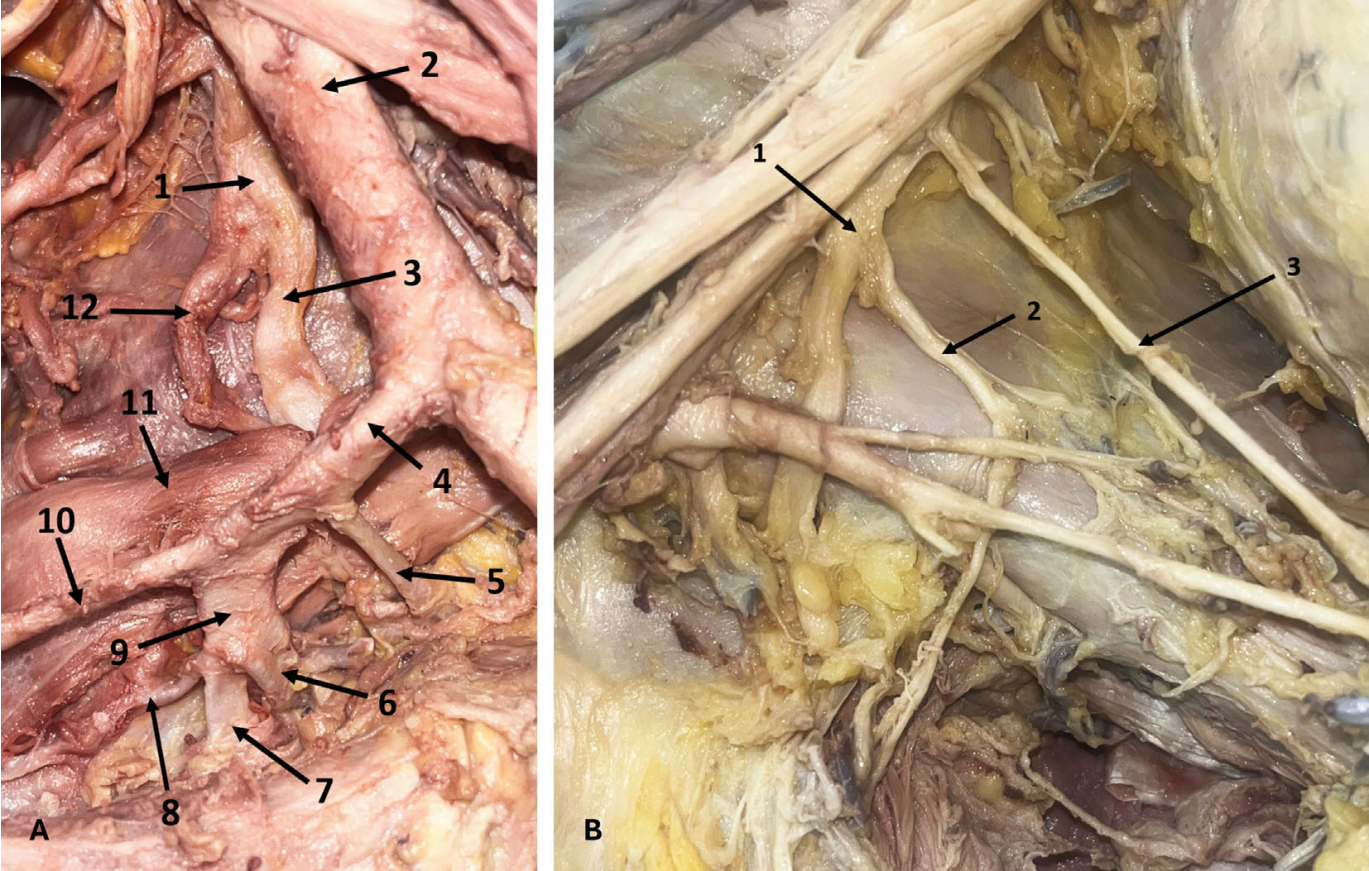
Variations in the AN and the LSSN. (A) Variations of the AN and accessory subscapularis muscle (left axilla): 1, AN; 2, AA; 3, AD of AN; 4, SSA; 5, PCHA; 6, CSA; 7, arterial branch to teres major; 8, arterial branch to teres minor; 9, CSA; 10, thoracodorsal artery; 11, accessory subscapularis muscle; 12, PD of AN. (B) Variation in the origin of the LSSN (right axilla): 1, AN; 2, LSSN; 3, thoracodorsal nerve. Abbreviations: AA, axillary artery; AD, anterior division; AN, axillary nerve; CSA, circumflex scapular artery; LSSN, lower subscapular nerve; PCHA, posterior circumflex humeral artery; PD, posterior division; SSA, subscapular artery.

In the right axilla of a 58‐year‐old male donor, the AN bifurcated into AD and PD 45.13 mm distal to its origin. The PD then bifurcated, and all three branches entered the QdrSp (Figure 9A). Another previously undescribed variation was observed in the right axilla of a 55‐year‐old male donor, in which the AN trifurcated, with the AD and a thin middle branch entering the QdrSp, and the PD passing through the TrSp (Figure 9B). In the left axilla of a 36‐year‐old male donor, the AN bifurcation was associated with an accessory SSM. Both divisions passed beneath the SSM, with the AD entering the QdrSp and the PD passing through the TrSp alongside the CSA—a combination of muscular and nerve variation not previously reported. Arterial variations were also noted (Figure 10A).

### 3.7. LSSN Variation

In the right axilla of a 64‐year‐old female donor, the LSSN originated from the AN 13.65 mm distal to its formation (Figure 10B).

### 3.8. TDN and PC Variations

**Figure 11:**
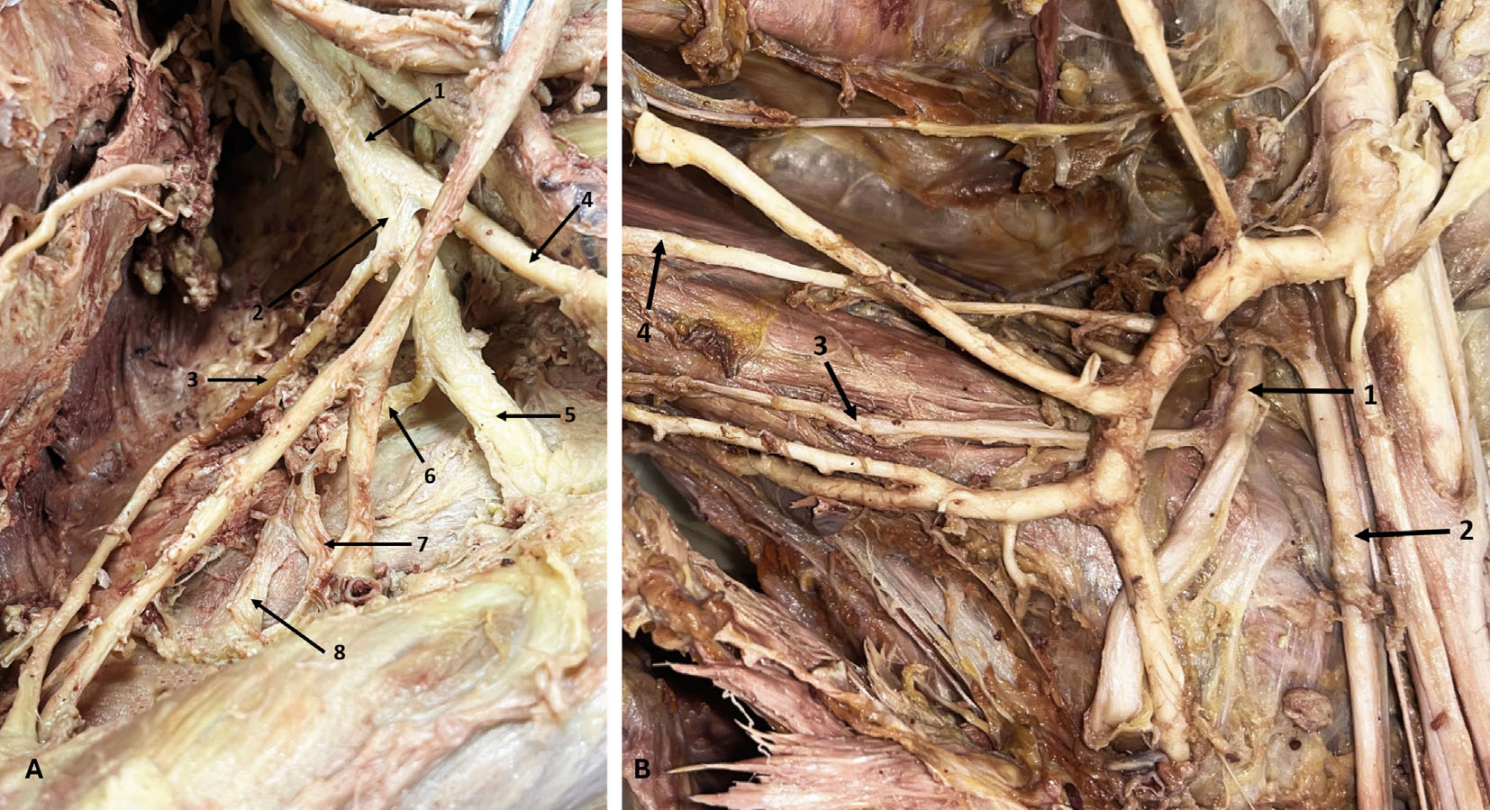
Variations of the TDN. (A) TDN originating from the AN (left axilla): 1, PC; 2, AN; 3, TDN; 4, RN; 5, AD of AN; 6, PD of AN; 7, a branch to teres major; 8, a branch to teres minor. (B) Accessory TDN (left axilla): 1, AN; 2, RN; 3, accessory TDN; 4, TDN. Abbreviations: AD, anterior division; AN, axillary nerve; PC, posterior cord; PD, posterior division; RN, radial nerve; TDN, thoracodorsal nerve.

Two TDN variations were found. In the left axilla of a 63‐year‐old male donor, the TDN originated from the AN (Figure 11A). In the left arm of a 78‐year‐old female donor, an accessory TDN also arose from the AN, 17.61 mm distal to the formation of the latter (Figure 11B)—accessory TDN has rarely been reported, and not from this origin in previous literature. On the same side, the PC bifurcated into the RN and TDN, with the AN branching from the PC at a higher level.

**Figure 12:**
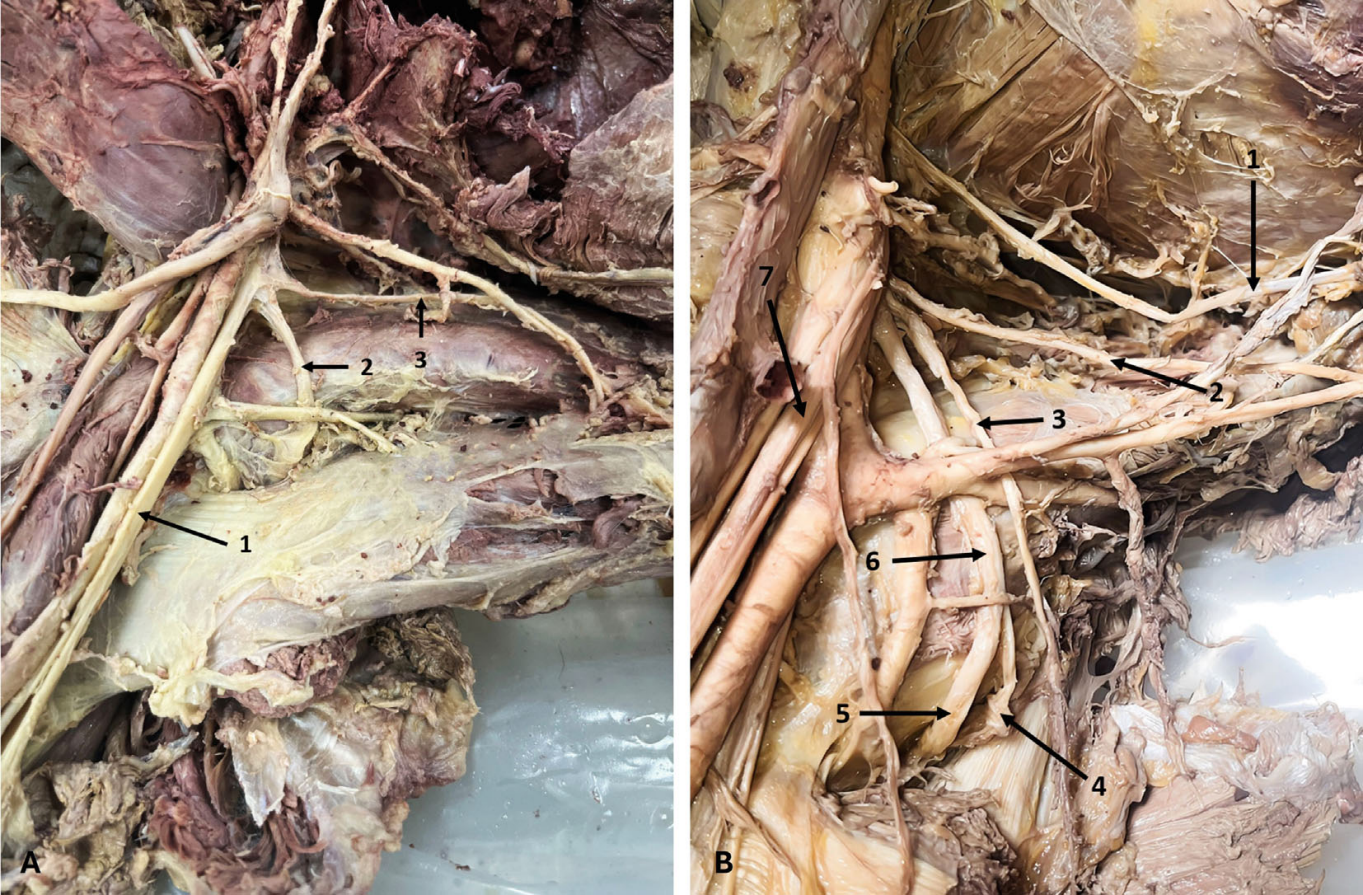
Variation in the PC. (A) Trifurcation of the PC (right axilla): 1, RN; 2, AN; 3, TDN. (B) Quadfurcation of the PC (right axilla): 1, long thoracic nerve; 2, TDN; 3, LSSN; 4, PD of AN; 5, AD of AN; 6, AN; 7, RN. Abbreviations: AD, anterior division; AN, axillary nerve; LSSN, lower subscapular nerve; PC, posterior cord; PD, posterior division; RN, radial nerve; TDN, thoracodorsal nerve.

Two additional PC variations were observed. In the right axilla of a 63‐year‐old male donor, the PC trifurcated into the AN, RN, and TDN (Figure 12A). On the right side of a 78‐year‐old female donor, the PC quadfurcated into the AN, RN, TDN, and LSSN (Figure 12B). To our knowledge, quadfurcation of the PC has not been described in the literature before.

## 4. Discussion

Anatomical variations of the BP have been widely documented, but the infraclavicular portion—particularly within the axilla and upper arm—remains a key area for surgical, anesthetic, and diagnostic relevance. Our study identified several variations in the cords and terminal branches, some corresponding to previously reported patterns, while others appear to be unreported in the literature.

The prevalence of BP variability has been reported to range from 13.3% to 90% [2–6, 11, 22, 23, 26–29]. In our study, BP variations were present in 44.4% of all dissected plexuses, with variable nerve structures accounting for 88.9%. This is within the range reported by previous authors. While most studies describe BP variations as typically unilateral [2, 5, 6, 11, 18, 26–28], our findings contrast—most variations were bilateral.

### 4.1. MN

The reported incidence of MN variations ranges from 10% to 71.4% [2, 6, 8, 11, 18, 22–24, 26, 30–36]. We found four variable MNs (12.5%), consistent with this range. The most common variation involved MN formation by three roots—two LRs and one MR—with the additional LR originating from the LC. We observed this in three cases. In one case, the three roots of the MN were positioned between the main and aberrant AA; to our knowledge, this configuration has not been previously described.

Using the criteria of Buch‐Hansen [37], these were classified as additional LRs rather than CBs between the MCN and MN, as the proximal LR was thinner and originated higher than the MCN. This pattern occurs in 5%–90% of reported cases [2, 5, 8, 11, 18, 22–24, 26, 27, 33–36, 38], and our frequency (75% of MN variations) is consistent with prior reports. Other known MN formation patterns include the LR arising from the MCN or from the MT [18, 30, 31, 34, 36, 38], as well as a single root from the LC (< 0.1%) or multiple roots—up to four—from combinations of the LC and MC [8, 30–32, 34, 36, 38].

In the present study, one MN exhibited a low fusion of its roots in the proximal third of the arm (25% of the MN variations). This frequency is higher than the 1.67%–20.0% range reported by most authors [2, 26, 30, 31, 34], but close to the 60.5% reported by Behnejad et al. [38]. Pattanshetti et al. [2] similarly found more frequent fusions in the upper third of the arm (13.33%) compared to the middle third (6.67%), in agreement with our data.

Several studies have reported CBs from the LC to the roots of the MN, with CBs from the LC to the MR being more commonly observed [6, 24, 28, 39, 40]. The meta‐analysis by Behnejad et al. [38] confirmed interconnections between the MN roots, including cases where the MR received a CB from the AD of the MT. We documented an exceptionally rare pattern: a CB between the MC and the LR of the MN crossing anterior to the AA. To the best of our knowledge, only one similar case has been reported in the literature [6]. This extremely rare arrangement has potential surgical relevance due to its proximity to the AA.

### 4.2. MCN

The MCN is often cited as the most variable nerve [20]. Multiple classifications describe its variations and CBs with the MN [9, 41–47].

Le Minor [41] classified MCN variations into five types: Type 1, no CB between the MCN and the MN, with no variation of the MCN; Type 2, a CB between the MCN and MN; Type 3, the LR of the MN originates from the MCN; Type 4, the MCN branches off from the MN; and Type 5, absence of the MCN or fusion of the MCN with the MN. Venieratos and Anagnostopoulou [43] proposed three types: Type 1, CB proximal to the MCN entry into CBM; Type 2, CB distal to CBM; and Type 3, the MCN does not pierce the CBM. Choi et al. [9] further classified the CBs between the MCN and the MN based on their number: Pattern 1, fusion between the MCN and MN; Pattern 2, a single CB between the MCN and MN, subdivided into Pattern 2a—a single branch that may be located proximal to the CBM, from within the muscle, distal to the muscle, or cases where the MCN does not pierce the CBM—and Pattern 2b, two or three branches from the MCN forming one CB; and Pattern 3, two CBs. Loukas and Aqueelah [44] described four types: Type 1, CBs proximal to the MCN entry into CBM; Type 2, CBs distal to the MCN entry into CBM (from within the CBM); Type 3, MCN does not enter the CBM; and Type 4, a combination of the other types.

In our study, we observed five CBs between the MCN and MN. Three CBs arose from MCNs that did not pierce the CBM, while two were proximal to the MCN entry point into the CBM. We also documented a fusion between the MCN and MN, corresponding to Type 5 in Le Minor′s classification [41] and Pattern 1 in Choi′s [9]. By Venieratos and Anagnostopoulou′s [43] and Loukas and Aqueelah′s [43] systems, two CBs were Type 1, and three were Type 3; all were Pattern 2a in Choi′s classification [9].

One of our findings was MCN–MN fusion. Literature describes similar cases either as “fusion” or “absence” of the MCN. Following Choi et al. [9] and Guerri‐Guttenberg and Ingolotti [45], we classified ours as fusion, not absence, since motor branches to the BBM and BM were present before continuation as the LCNFA. The reported incidence of such fusion ranges from 6.5% to 19.2% [9, 38]. Similar cases have been documented as fusion by some authors [9, 21, 40, 48–50] and as absence by others [51, 52].

Another recognized variation of the MCN occurs when the nerve does not pierce the CBM. Reported incidences range from 3% to 29.6% [9, 15, 24, 27, 29, 44–47, 53]. In the present series, three out of six MCNs (50%) did not pierce the CBM, representing a markedly higher frequency than most previous reports. This difference may reflect our small sample size or population‐specific anatomical characteristics.

The most common MCN variation is the presence of a CB from the MCN to the MN [38, 43]. Reported frequencies vary from 13.7% to 63.57% per donor [9, 42–44, 53] and from 5% to 53.6% per BP [9, 10, 15, 43–47, 54, 55]. Our frequency (13.9%) falls within this range. In all examined limbs, the pattern consisted of a single CB—the most common configuration reported in the literature [9, 10, 14, 15, 43–46, 54]. Double CBs are less frequent (2%–10.7%) [9, 38, 45, 55–57], and we did not observe them.

Regarding CB position relative to the MCN entry into the CBM, previous studies disagree: some report predominantly proximal CBs [44, 45, 47], while others note distal CBs as more frequent [9, 14, 43, 54]. Sirico et al. [20] found that 45.97% of CBs were distal. Our findings diverge from this pattern, as most CBs arose from MCNs that did not pierce the CBM. The remaining two CBs were proximal, matching the observations of other authors [44, 45, 47]. Consistent with the literature [9, 44, 47, 54, 55], three CBs were unilateral and two bilateral.

Some authors describe CBs arising from the MN and joining the MCN, with reported incidences of 2.8%–30.1% [2, 6, 10, 46, 54, 55], but we did not identify such cases.

One particularly rare variation in our series involved MCN that did not pierce the CBM, gave off a CB to the MN, and then bifurcated into two well‐defined divisions: LD and MD. While Patel and Smith [24] reported nine cases where the MCN gave off multiple proximal branches before entering the CBM, none resembled our configuration. To our knowledge, this precise arrangement has not been previously documented.

### 4.3. UN

CBs between the MN and the UN in the forearm and hand are well‐documented [58–60]. However, reports of such connections in the axillary region and upper arm are scarce. The most common UN variation in these regions involves its formation by two roots—LR and MR. The LR of the UN may arise from a CB originating from the LC, with a reported incidence of 2.0%–30% [6, 8, 18, 22, 24, 26, 61]. Alternatively, the LR of the UN can develop from a CB originating from the LR of the MN [6, 38, 48, 62, 63].

Gopal and Singh [28] described a case where the LC communicated with both the MR of the MN and the UN. Elzawawy et al. [3] reported a CB between the MR of the MN and the UN. In their review, Benes et al. [8] identified three possible origins of the UN: from the MC with an accessory branch from the LC (2.0%), from the MC with two accessory branches from the LC (< 0.1%), and from the MC with an accessory branch from the PC (< 0.1%). Hassan and Jan [5] reported a double CB between the LR of the MN and the UN, while Taib et al. [64] documented a CB between the MN and the UN that closely resembles the variation we observed. This supports the rarity of such a configuration in the proximal upper limb and underscores its potential clinical significance.

### 4.4. RN

The RN is generally regarded as the least variable nerve, and its variations are rare [8, 26]. Several authors have reported a high division of the RN into two branches, with an incidence ranging from 1.67% to 44% [2, 65–68]. In some cases, both divisions entered the TrI, with one continuing as the main RN and the other providing all muscular and sensory branches to the arm [2, 65–67]. In other cases, the AD continued as the main RN, while the PD supplied the TrM [67, 69].

Raghavendra et al. [69] found high division in 44% of 50 upper limbs. The RN divided into AD and PD in 59.1% of the cases or into LD and MD in 40.9%. The AD or the LD continued as the main RN, while the PD or MD innervated the TrM. They also reported that most divisions occurred in the axilla (77.3%), with only 22.7% in the TrI. Churikov et al. [66] observed high division into MD and LD in 27.5% of cases. The MD continued as the main RN without giving branches in the upper and middle thirds of the arm. The LD innervated the posterior arm muscles and provided sensory innervation to the posterior arm and forearm. Ramasamy and Kalaivanan [67] described a similar pattern, with the AD continuing as the main RN and the PD supplying the TrM.

In present study, we identified three RNs forming two divisions and two RNs forming three divisions. In the bifurcation cases, one division entered the TrI as the main RN, while the other supplied the TrM. In the trifurcation cases, two divisions entered the TrI, and the third division supplied the TrM. The overall incidence of this variation was 15.6%, consistent with the upper range of previous reports. The presence of RN trifurcation is particularly uncommon, with only two case reports describing trifurcation of the RN within the spiral groove [70, 71]. Although similar, our cases differ in that the RN trifurcated above the TrI and radial groove. This finding highlights the complexity of possible branching patterns in what is often considered the least variable major nerve of the BP.

### 4.5. AN

Variations in the branching pattern of the AN are relatively rare in the literature. In the present study, most variable ANs divided into AD and PD above the QdrSp. In several cases, both divisions passed through the QdrSp. Previous studies have shown that the AN most often divides into AD and PD within the QdrSp [72–76]. Uz et al. [77] reported that in 3 out of 30 cases (10%), the AN divided at the inferolateral border of SSM, and in 1 case (3.3%), it branched while coursing over the SSM. Jaishree and Ashwini [78] described AN bifurcation above the QdrSp, with both branches entering the space. Pal et al. [79] reported AN division within the QdrSp in 88% of cases and above the space in 12%.

We observed one trifurcation of the AN, with the AD and a middle branch passing through the QdrSp and the PD passing through the TrSp, and two ANs in which the PD passed through the TrSp together with the CSA. This appears to be the first documented report of AN trifurcation with an unusual course of its divisions, as well as the PD of the AN traveling with the CSA through the TrSp.

In our series, two ANs showed a PD that supplied both the TMi and TMj. Muthoka et al. [80] noted that the LSSN can be absent in 12% of cases, which may explain why the AN occasionally supplied the TMj. Pandey et al. [81] reported a bilateral variation where the AN gave a branch to the TMj muscle. Oluoch et al. [75] found that the main trunk of the AN supplied the TMj in 28.57% of the cases. However, our finding is novel—in these cases, the posterior branch of the AN, not the main trunk, innervated both TMi and TMj. To our knowledge, this specific pattern has not been previously described.

In one donor, the AN variation was accompanied by a muscular variation—an accessory SSM located anterior to the AN. This accessory muscle has been described by other authors [81–83], but its coexistence with AN branching variation is rare. Such a configuration may predispose the AN to compression, potentially resulting in entrapment syndrome. Clinical symptoms could involve dysfunction of the shoulder joint, deltoid muscle, TMi, and TMj, particularly when the TMj is innervated by a branch of the AN [81–83].

### 4.6. LSSN

We observed one case where the LSSN arose from the AN. Ballesteros and Ramirez [84] reported that nearly 70% of the LSSN cases had a variable origin, with the AN being the most common source (54.4%). The reported incidence of this variation ranges from 3.3% to 57.3% [18, 80, 84, 85]. Some studies have described a common trunk (CT) between the AN and LSSN, occurring in 5%–17.2% of cases [8, 29, 86]. In the study by Bustamante Aliste et al. [87], the LSSN branched from a CT with the TDN in 10% of the limbs and directly from the AN in 90%, with no cases arising from the PC. In our series, the LSSN variation accounted for 3.1%, which is at the lower end of reported frequencies.

### 4.7. TDN

We also identified two variations of the TDN. In one donor, the TDN arose from the AN, and in another, an accessory TDN originated from the AN. The TDN typically branches from the PC between the USSN and LSSN [88]. Al‐Hubaity et al. [26] found this typical pattern in only 41.7% of 60 limbs. Other origins included the TDN as the last branch of the PC (21.7%), at the same level as the AN (20%), and as a CT with the AN (16.7%). Benes et al. [8] documented multiple origins: from the PC (90.1%); from the PD of the ST or the MT (< 0.1% each); from CTs involving USSN, LSSN, or AN (< 4.3% combined); and from the RN (0.4%). The incidence of the TDN originating from the AN ranges from 8.9% to 22.9% [18, 24, 80, 84, 89, 90]. In the present study, both variable TDNs arose from the AN with an incidence of 6.2%, which is lower than most previous reports.

Importantly, the accessory TDN in our study originated from the AN rather than the PC, making it a particularly rare finding. Benes et al. [8] reported accessory TDNs in < 0.1%, all from the PC. Gaur et al. [91] described an extra branch from the PC running parallel to the TDN and supplying TMj and latissimus dorsi muscle. Pattanshetti et al. [2] observed accessory TDNs in 3.33% of cases, both from the PC. To our knowledge, ours is the first report of an accessory TDN arising from the AN.

### 4.8. PC

We identified three variations in the branching pattern of the PC. The first variation involved a high origin of the AN, which branched from the PC above the level of the TDN. The PC then bifurcated into the TDN and the RN. Al‐Hubaity et al. [26] reported that in 21.7% of cases, the TDN is the last branch of the PC before it continues as the RN. A similar pattern was also noted by Pandey et al. [81].

We also observed one instance of PC trifurcation and one instance of PC quadfurcation. Gaur et al. [91] found that in 2% of cases, the PC gave off all branches from a single point, and in 8%, it divided into two divisions—one continuing as the RN, and the other giving rise to all the branches and then continuing as the AN. In their review, Bhosale and Mallshetty [90] reported a CT between the AN and the TDN, which resembles our trifurcation. However, we identified our case as a true trifurcation of the PC rather than a CT. Patel and Smith [24] also described cases where the TDN branched from the same point as the RN and AN. To our knowledge, this is the first report of a quadfurcation of the PC. No previous literature describes the PC dividing into the AN, RN, TDN, and LSSN as separate branches from a single point. This finding adds a previously undocumented pattern to the known spectrum of PC variations.

### 4.9. Clinical Significance

The BP is often affected by anatomical variations, many of which have important clinical implications [3–5]. Knowledge of these variations is critical to prevent misdiagnoses, failed anesthetic blocks, and inadvertent nerve or vascular injuries [6, 7, 11, 54]. Variations in the infraclavicular part of the BP can produce unexpected symptoms such as sensory loss, pain, nerve palsy, or vascular issues due to altered relationships with nearby muscles and vessels [18, 72].

The MCN and MN are especially prone to variations. Accessory roots of the MN may compress adjacent vessels [38]. When the MCN is absent or fused with the MN, lesions of the MN can produce deficits in the flexor muscles of the arm and/or the lateral cutaneous region of the forearm, complicating posttrauma evaluations [40, 45]. Depending on its location, a CB between the MCN and MN can cause unexpected neurological signs or even ischemic pain and arterial insufficiency of the AA if one of the nerves is injured [38, 45]. In the present study, we observed a previously undescribed CB between the MC and the LR of the MN crossing anterior to the AA, which could have significant surgical and anesthetic relevance.

RN variations can alter the success of reconstructive surgeries [66, 69]. In high division cases, failure to identify both trunks during surgery may leave one unrepaired, causing persistent deficits and reducing surgical success [66, 69]. We documented cases of RN bifurcation and trifurcation, patterns that may pose a risk during axillary or upper arm procedures.

The AN is highly susceptible to damage in shoulder trauma and surgery, including arthroscopy and plate fixation [73, 92]. Variations in its branching pattern may lead to serious motor and sensory impairments if injured [72, 73]. In the present series, we identified two previously unreported AN patterns—the PD passing through the TrSp alongside the CSA and a PD supplying both TMi and TMj. Both patterns could affect surgical access to the posterior scapular region and increase entrapment risk.

The TDN and LSSN may originate from the AN, making them vulnerable in posterior axillary wall injuries [84]. Furthermore, injuries to the PC can result in sensory loss, pain, or paresis, with variations complicating both diagnosis and treatment [84, 91]. We found an extremely rare accessory TDN arising from the AN—a pattern not previously documented in the literature as well as the first documented case of PC quadfurcation, along with a trifurcation, both of which could complicate nerve blocks, neurotization, or reconstructions in the axilla [80].

A detailed understanding of infraclavicular BP variations is essential for accurate clinical interpretation, safer anesthesia, and more effective surgical repair. Recognizing these rare and unique patterns—such as an MN with an accessory LR between the main and aberrant AA, a CB between the MC and LR of the MN crossing the AA, a CB between the UN and MN in the upper arm, bifurcation of the MCN into LD and MD, trifurcation of the RN above the TrI, trifurcation of the AN with the AD and a thin middle branch entering the QdrSp and the PD passing through the TrSp, PD of the AN through the TrSp with CSA, AN branch to TMj and TMi, variable AN with accessory SSM, accessory TDN from the AN, and PC quadfurcation—can help clinicians avoid iatrogenic injury and improve patient outcomes.

This study has certain limitations. As a cadaveric morphological investigation, it cannot establish functional or clinical correlations, and the sample size was limited by specimen availability. In addition, no genetic analyses or in vivo imaging were performed. Future studies could integrate genetic assessments, such as single‐nucleotide polymorphism and copy number variation analyses, with advanced imaging modalities to correlate morphological variations of the BP with developmental, functional, and clinical outcomes. Such multidisciplinary approaches may provide deeper insights into the underlying causes of anatomical diversity and its surgical and anesthetic implications.

## 5. Conclusion

A thorough understanding of BP variations is essential for accurate diagnosis, safe regional anesthesia, and successful surgical interventions. In contrast to most literature, our study found AN variations to be the most common, with bilateral variations more frequent than unilateral ones and often accompanied by arterial variations. We also documented several rare and previously undescribed patterns. These findings underscore the remarkable anatomical diversity of the infraclavicular BP and highlight the need for awareness of such variations to minimize iatrogenic injury. Further research with larger samples is warranted to validate these observations.

**Nomenclature**
BP: brachial plexus
BPs: brachial plexuses
ST: superior trunk
MT: middle trunk
IT: inferior trunk
LC: lateral cord
MC: medial cord
PC: posterior cord
AA: axillary artery
CSA: circumflex scapular artery
MCN: musculocutaneous nerve
MCNs: musculocutaneous nerves
MN: median nerve
MNs: median nerves
UN: ulnar nerve
AN: axillary nerve
ANs: axillary nerves
RN: radial nerve
RNs: radial nerves
TDN: thoracodorsal nerve
LSSN: lower subscapular nerve
USSN: upper subscapular nerve
LCNFA: lateral cutaneous nerve of forearm
LR: lateral root
LRs: lateral roots
AD: anterior division
ADs: anterior divisions
PD: posterior division
PDs: posterior divisions
LD: lateral division
MD: medial division
CB: communicating branch
CBs: communicating branches
QdrSp: quadrangular space
TrSp: triangular space
TrI: triangular interval
CBM: coracobrachialis muscle
BBM: biceps brachii muscle
BM: brachialis muscle
TMi: teres minor muscle
TMj: teres major muscle
TrM: triceps muscle
SSM: subscapularis muscle
CT: common trunk

## Conflicts of Interest

The authors declare no conflicts of interest.

## Author Contributions

E.B.: dissections, measurements, data analysis, manuscript writing, reviewing, and editing; J.C.D.: dissection; P.H.M.: dissection; N.U.: manuscript reviewing and editing.

## Funding

No funding was received for this manuscript.

## Data Availability

We confirm that the data supporting the findings of this study are available within the article.
